# Editorial Comment to Differences in 5-Aminolevulinic Acid-Induced Hemodynamic Changes between Patients Undergoing Neurosurgery and Urological Surgery

**DOI:** 10.31662/jmaj.2021-0166

**Published:** 2021-09-27

**Authors:** Hideo Fukuhara

**Affiliations:** 1 Departments of Urology, Kochi Medical School, Nankoku, Japan

**Keywords:** 5-aminolevulinic acid, Hypotension, photodynamic diagnosis

In Japan, 5-aminolevulinic acid-based photodynamic diagnosis (ALA-PDD) is an essential tool for complete resection in glioma and bladder cancer. The benefit of ALA-PDD is the visualization of tumor margin during operation. In bladder cancer, ALA-PDD is especially useful for the detection of tiny and flat lesions. Additional detection rate, which cannot detect tumor by conventional white light is between 10% and 30% ^(1), (2)^. ALA-PDD for bladder cancer has improved diagnostic accuracy has decreased recurrence rate after transurethral resection of bladder tumor (TURBT). In Japan, ALA is orally administrated before operation in glioma and bladder cancer. Additionally, hypotension is a well-known and important adverse effect.

Nohara et al reported induction of intraoperative hypotension by ALA in bladder cancer ^(3)^. They reported that general use of RAS inhibitor and general anesthesia were risk factors for ALA-induced hypotension.

Chung et al reported the relation between ALA-dose and hypotension in glioma ^(4)^. They concluded that ALA-dose was not related to ALA-induced hypotension; however, they identified antihypertensive drug as the risk factor of hypotension.

In general, severe type of hypotension by ALA shows catecholamine resistant character. The mechanism of ALA-mediated hypotension is unknown. Archor et al. hypothesized the vascular dilation by releasing a lot of nitric oxide.

The authors suggested the difference of ALA-induced hypotension between neurosurgery and urological surgery ^(5)^. The authors focused on hemodynamics in each surgery and found that hemodynamic parameters were not significantly different between ALA-pretreated and nonpretreated neurosurgery-patients. The blood pressure (BP) in urological surgery was significantly lower than those in nonpretreated patients. The low BP was observed for 9h after ALA administration. In addition, HR values in urological surgery were higher than those in neurosurgery. ALA-PDD in urological surgery may be more susceptible to hemodynamic effects than those in neurosurgery. They concluded that ALA-induced hemodynamics might differ between neurosurgery and urological surgery.

The limitations of this study are small sample volume and retrospective nature. Further, large sample study will indicate the appropriate risk factors of intraoperative hypotension by 5-aminolevulinic acid.

## Article Information

### Conflicts of Interest

None
